# Supplementary material for: Multiple timescales of sensory-evidence accumulation across the dorsal cortex
Source: eLife. 2022 Jun 16;11:e70263. doi: 10.7554/eLife.70263 (PMC9203055; doi:10.7554/eLife.70263)
Supplement: Figure 1—source data 1. — Last line shows the number of unique mice and trials across all experiments, as conditions were partially overlapping for a given mouse and behavioral session. [file elife-70263-fig1-data1.docx]

| **Region** | **Epoch** | **Num. control trials** | **Num. laser trials** | **Num. mice** | **Num. sessions** | **Total trial count** |
| --- | --- | --- | --- | --- | --- | --- |
| V1 | cue, 1^st^ quarter (0 – 50 cm) | 22,582 | 725 | 9 | 200 | 23,307 |
| V1 | cue, 2^nd^ quarter (50 – 100 cm) | 22,465 | 727 | 9 | 200 | 23,192 |
| V1 | cue, 1^st^ half (0 – 100 cm) | 20,061 | 549 | 8 | 160 | 20,610 |
| V1 | cue, 3^rd^ quarter (100 – 150 cm) | 22,417 | 717 | 9 | 196 | 23,134 |
| V1 | cue, 2^nd^ half (100 – 200 cm) | 20,805 | 594 | 8 | 165 | 21,399 |
| V1 | delay (200 - 300 cm) | 20,388 | 614 | 9 | 165 | 21,002 |
| mV2 | cue, 1^st^ quarter (0 – 50 cm) | 19,087 | 620 | 9 | 168 | 19,707 |
| mV2 | cue, 2^nd^ quarter (50 – 100 cm) | 19,213 | 618 | 9 | 171 | 19,831 |
| mV2 | cue, 1^st^ half (0 – 100 cm) | 15,036 | 382 | 4 | 125 | 15,418 |
| mV2 | cue, 3^rd^ quarter (100 – 150 cm) | 19,136 | 622 | 9 | 168 | 19,758 |
| mV2 | cue, 2^nd^ half (100 – 200 cm) | 15,113 | 372 | 4 | 122 | 15,485 |
| mV2 | delay (200 - 300 cm) | 15,647 | 381 | 6 | 127 | 16,028 |
| PPC | cue, 1^st^ quarter (0 – 50 cm) | 16,644 | 428 | 7 | 140 | 17,072 |
| PPC | cue, 2^nd^ quarter (50 – 100 cm) | 16,735 | 456 | 8 | 143 | 17,191 |
| PPC | cue, 1^st^ half (0 – 100 cm) | 9,176 | 655 | 10 | 64 | 9,831 |
| PPC | cue, 3^rd^ quarter (100 – 150 cm) | 16,698 | 453 | 8 | 142 | 17,151 |
| PPC | cue, 2^nd^ half (100 – 200 cm) | 7,592 | 277 | 6 | 53 | 7,869 |
| PPC | delay (200 - 300 cm) | 9,281 | 671 | 8 | 70 | 9,952 |
| RSC | cue, 1^st^ quarter (0 – 50 cm) | 16,570 | 448 | 7 | 141 | 17,018 |
| RSC | cue, 2^nd^ quarter (50 – 100 cm) | 16,762 | 450 | 8 | 144 | 17,212 |
| RSC | cue, 1^st^ half (0 – 100 cm) | 11,816 | 938 | 10 | 86 | 12,754 |
| RSC | cue, 3^rd^ quarter (100 – 150 cm) | 16,726 | 461 | 8 | 144 | 17,187 |
| RSC | cue, 2^nd^ half (100 – 200 cm) | 13,947 | 1,147 | 11 | 100 | 15,094 |
| RSC | delay (200 - 300 cm) | 10,339 | 867 | 9 | 76 | 11,206 |
| Posterior | cue, 1^st^ quarter (0 – 50 cm) | 20,411 | 598 | 7 | 159 | 21,009 |
| Posterior | cue, 2^nd^ quarter (50 – 100 cm) | 20,586 | 624 | 7 | 162 | 21,210 |
| Posterior | cue, 1^st^ half (0 – 100 cm) | 15,070 | 372 | 4 | 123 | 15,442 |
| Posterior | cue, 3^rd^ quarter (100 – 150 cm) | 20,549 | 610 | 7 | 161 | 21,159 |
| Posterior | cue, 2^nd^ half (100 – 200 cm) | 14,793 | 327 | 4 | 123 | 15,120 |
| Posterior | delay (200 - 300 cm) | 14,843 | 370 | 4 | 119 | 15,213 |
| mM2 | cue, 1^st^ quarter (0 – 50 cm) | 22,530 | 728 | 9 | 199 | 23,258 |
| mM2 | cue, 2^nd^ quarter (50 – 100 cm) | 22,484 | 734 | 9 | 198 | 23,218 |
| mM2 | cue, 1^st^ half (0 – 100 cm) | 20,315 | 602 | 8 | 164 | 20,917 |
| mM2 | cue, 3^rd^ quarter (100 – 150 cm) | 22,560 | 719 | 9 | 199 | 23,279 |
| mM2 | cue, 2^nd^ half (100 – 200 cm) | 21,333 | 649 | 9 | 169 | 21,982 |
| mM2 | delay (200 - 300 cm) | 20,129 | 618 | 8 | 162 | 20,747 |
| aM2 | cue, 1^st^ quarter (0 – 50 cm) | 16,672 | 453 | 7 | 142 | 17,125 |
| aM2 | cue, 2^nd^ quarter (50 – 100 cm) | 16,699 | 451 | 8 | 142 | 17,150 |
| aM2 | cue, 1^st^ half (0 – 100 cm) | 20,655 | 589 | 8 | 168 | 21,244 |
| aM2 | cue, 3^rd^ quarter (100 – 150 cm) | 16,731 | 451 | 8 | 143 | 17,182 |
| aM2 | cue, 2^nd^ half (100 – 200 cm) | 21,557 | 673 | 9 | 172 | 22,230 |
| aM2 | delay (200 - 300 cm) | 20,110 | 635 | 8 | 161 | 20,745 |
| M1 | cue, 1^st^ quarter (0 – 50 cm) | 14,539 | 396 | 7 | 124 | 14,935 |
| M1 | cue, 2^nd^ quarter (50 – 100 cm) | 14,561 | 400 | 7 | 126 | 14,961 |
| M1 | cue, 1^st^ half (0 – 100 cm) | 15,448 | 404 | 4 | 130 | 15,852 |
| M1 | cue, 3^rd^ quarter (100 – 150 cm) | 14,611 | 394 | 7 | 127 | 15,005 |
| M1 | cue, 2^nd^ half (100 – 200 cm) | 15,365 | 409 | 4 | 126 | 15,774 |
| M1 | delay (200 - 300 cm) | 15,280 | 404 | 4 | 125 | 15,684 |
| Frontal | cue, 1^st^ quarter (0 – 50 cm) | 20,555 | 623 | 7 | 161 | 21,178 |
| Frontal | cue, 2^nd^ quarter (50 – 100 cm) | 20,553 | 622 | 7 | 162 | 21,175 |
| Frontal | cue, 1^st^ half (0 – 100 cm) | 14,951 | 385 | 4 | 122 | 15,336 |
| Frontal | cue, 3^rd^ quarter (100 – 150 cm) | 20,444 | 636 | 7 | 157 | 21,080 |
| Frontal | cue, 2^nd^ half (100 – 200 cm) | 14,964 | 392 | 4 | 124 | 15,356 |
| Frontal | delay (200 - 300 cm) | 15,131 | 385 | 4 | 122 | 15,516 |
| **Total unique count** | | 108,940 | 29,825 | 28 | 929 | 138,765 |
